# A Deep Autoencoder Compression-Based Genomic Prediction Method for Whole-Genome Sequencing Data

**DOI:** 10.3390/biology14111622

**Published:** 2025-11-19

**Authors:** Hailiang Song, Tian Dong, Wei Wang, Xiaoyu Yan, Chenfan Geng, Song Bai, Hongxia Hu

**Affiliations:** 1 Fisheries Science Institute, Beijing Academy of Agriculture and Forestry Sciences & Beijing Key Laboratory of Fisheries Biotechnology, Beijing 100068, China; songhl0317@163.com (H.S.); yinghua328@163.com (T.D.); raywang8848@163.com (W.W.); yanxiaoyu21@126.com (X.Y.); gengchenfishery@163.com (C.G.); baisong@baafs.net.cn (S.B.); 2 Key Laboratory of Sturgeon Genetics and Breeding, Ministry of Agriculture and Rural Affairs, Hangzhou 311799, China

**Keywords:** deep autoencoder, data compression, genomic prediction, whole-genome sequencing

## Abstract

Genomic prediction using whole-genome sequencing (WGS) data is challenged by the imbalance between a limited sample size (*n*) and an extensive number of single-nucleotide polymorphisms (SNPs) (*p*), where *n* ≪ *p*. The high dimensionality of WGS data also increases computational demands, limiting its practical application. In this study, we introduce DAGP, a novel method that integrates deep autoencoder compression to reduce WGS data dimensionality by over 99% while preserving essential genetic information. This compression significantly improves computational efficiency, facilitating the effective use of high-dimensional genomic data. Our results demonstrated that DAGP, when combined with the genomic best linear unbiased prediction (GBLUP) method, maintained prediction accuracy comparable to WGS data, even at reduced marker densities of 50 K for sturgeon and 20 K for maize. Furthermore, integrating DAGP with Bayesian and machine learning models improved genomic prediction accuracy over traditional WGS-based GBLUP, with an average gain of 6.05% and 5.35%, respectively. DAGP provides an efficient and scalable solution for genomic prediction in species with large-scale genomic data, offering both computational feasibility and enhanced prediction performance.

## Simple Summary

Predicting the genetic potential of animals and plants from their DNA can greatly accelerate breeding for traits such as growth, yield, or disease resistance. However, whole-genome sequencing produces extremely large datasets that are difficult and expensive to analyze. This study introduces a new method called deep autoencoder-based genomic prediction (DAGP), which uses artificial intelligence to compress genetic data by more than 99% while retaining the most important biological information. This approach makes it possible to process and analyze genetic data much faster without losing accuracy. When tested in both sturgeon and maize, the method achieved prediction accuracy equal to or higher than that of conventional approaches, while requiring only a fraction of the data and computing resources. By providing a faster, more efficient, and scalable way to use full DNA information, this method can help breeders and scientists make better-informed decisions, improve the efficiency of selective breeding, and support sustainable food production across a wide range of species.

## 1. Introduction

Genomic selection (GS), first proposed by Meuwissen et al. [1], is a method for genetic evaluation in animal breeding that leverages genome-wide single-nucleotide polymorphism (SNP) markers. It operates under the assumption that at least one SNP is in linkage disequilibrium (LD) with each quantitative trait locus (QTL), allowing for the comprehensive tracking of genetic variation affecting a trait. This enables accurate estimation of an individual’s genomic estimated breeding value (GEBV), a key component of GS. GEBV estimation methods can be categorized into three groups: BLUP-based, Bayesian, and machine learning methods. BLUP-based methods, such as genomic BLUP (GBLUP) [2], construct a genomic relationship matrix using genetic data from reference and candidate populations and solve mixed model equations to estimate GEBVs. Bayesian methods, including BayesA and BayesB [1], estimate marker effects through prior and posterior distributions, using sampling techniques like Markov Chain Monte Carlo (MCMC) to derive GEBVs. Machine learning methods, such as support vector regression (SVR) and random forest (RF) [3], are particularly effective at capturing complex genotype-phenotype-environment interactions, offering a flexible approach to genomic prediction.

BLUP-based methods have driven breeding progress in GS, primarily by capturing linear relationships between genotype and phenotype. However, they typically assume that all markers follow the same distribution and are unable to effectively explain the nonlinear effects of complex traits [4]. In contrast, Bayesian methods assume that genetic variation is influenced by a subset of SNPs, allowing for different effect distributions and providing an advantage in modeling complex traits [1]. Similarly, machine learning methods can capture nonlinear relationships, further improving prediction accuracy for these traits [3]. As a result, Bayesian and machine learning methods offer greater potential than BLUP-based models for enhancing genomic prediction. With advancements in whole-genome sequencing (WGS) technology and decreasing costs, WGS data has become widely used in animal and plant breeding [5,6,7,8]. However, the vast number of markers—often in the tens of millions—poses computational challenges, limiting the applicability of Bayesian and machine learning methods in genomic prediction. This imbalance between the limited number of samples (*n*) and the extremely large number of SNPs (*p*), where *n* ≪ *p*, results in high computational demands and potential overfitting. Therefore, developing efficient data compression algorithms that minimize information loss while fully leveraging the strengths of Bayesian and machine learning approaches is crucial for improving genomic prediction accuracy.

In this study, we developed DAGP, a deep autoencoder compression-based genomic prediction method designed to reduce WGS data dimensionality while enhancing prediction accuracy. DAGP integrates GBLUP, Bayesian, and machine learning methods, leveraging their respective strengths for genomic prediction. For its general application, the efficiency of the proposed method was evaluated using real data from sturgeon and maize datasets.

## 2. Materials and Methods

### 2.1. DAGP Framework

**Figure 1:**
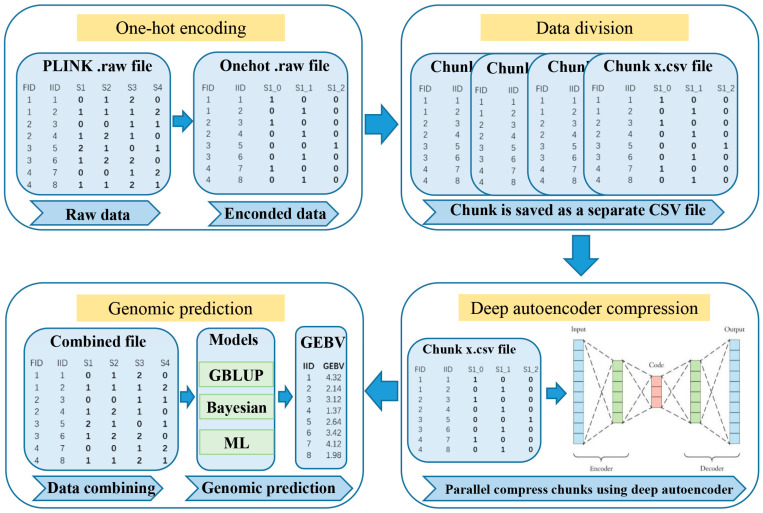
The framework of DAGP. DAGP consists of genotype encoding using one-hot encoding, data division into manageable chunks, and deep autoencoder compression for dimensionality reduction. The compressed data is then combined for genomic prediction using GBLUP, Bayesian, and machine learning (ML) methods, with performance evaluation based on prediction accuracy, bias, mean squared error (Mse) and mean absolute error (Mae).

DAGP is a genomic prediction framework designed to enhance genomic prediction accuracy. The overall framework of DAGP is illustrated in Figure 1.

#### 2.1.1. One-Hot Encoding and Data Division

Genotype data, typically represented by discrete values (0, 1, 2) corresponding to different alleles, was transformed into a binary vector format using one-hot encoding. Specifically, the value 0 was encoded as [1, 0, 0], 1 as [0, 1, 0], and 2 as [0, 0, 1]. This transformation enables the model to treat genetic variations as independent binary features, facilitating downstream analysis. To optimize memory usage and efficiently process large-scale datasets, the data was divided into smaller chunks. Missing or erroneous values were excluded during encoding to ensure data integrity. Parallelized encoding was performed using the joblib library, which split the data into multiple partitions and utilized multi-core processing for faster computation. The resulting encoded partitions were then concatenated to form the complete one-hot encoded dataset.

Following encoding, the genotype data was divided into smaller, manageable chunks. The dataset was split into a predefined number of parts, each containing an approximately equal number of SNPs, ensuring efficient parallel processing in subsequent stages of the DAGP method. Any remaining SNPs, resulting from an uneven division, were allocated to the last chunk to ensure full utilization of the data. Each chunk was saved as a separate CSV file, facilitating efficient storage, retrieval, and further analysis.

#### 2.1.2. Deep Autoencoder Compression

Following the one-hot encoding and data division steps, the WGS data was compressed using a deep autoencoder model [9,10]. This model was designed to reduce the dimensionality of the encoded genotype data, capturing essential genetic information while minimizing redundancy. The autoencoder architecture consisted of two symmetrical components: the encoder and the decoder. (1) Encoder network $h(x_{i})$, given an input $x_{i}\in R^{d}$, the encoder transformed $x_{i}$ into a compressed hidden representation ${h(x_{i})}^{\left(l+1\right)}$ (Equation (1)). (2) Decoder network $x_{i}^{\prime }$, the decoder reconstructed the input data from the hidden representation ${h(x_{i})}^{\left(l+1\right)}$ (Equation (2)).

(1) $${h\left(x_{i}\right)}^{\left(l+1\right)}=f\left(W^{\left(l\right)}x_{i}^{\left(l\right)}+b^{\left(l\right)}\right)$$

(2) $$x_{i}^{\prime \left(l\right)}=g\left(W^{\prime \left(l\right)}{h\left(x_{i}\right)}^{\left(l+1\right)}+b^{\prime \left(l\right)}\right)$$

In this architecture, f and g denote the activation functions for the encoder and decoder, respectively, while $W^{\left(l\right)}$ and $W^{\prime \left(l\right)}$ are the weight matrices, and $b^{\left(l\right)}$ and $b^{\prime (l)}$ are the bias vectors.

The activation function used for all layers, except the middle and decoder layers, was rectified linear unit (ReLU), which was defined as:

(3) $$ReLU\left(x\right)=\max\left(0,x\right)$$

For the middle and decoder layers, we used the sigmoid activation function, which maps the output to the range [0, 1]:

(4) $$\sigma \left(x\right)=\frac{1}{1+e^{-x}}$$

The model’s performance was optimized by minimizing the reconstruction error, which was computed using the mean squared error (MSE) function:

(5) $$MSE\left(x,x^{\prime }\right)=\frac{1}{n}\sum_{i=1}^{n}{\left\|x_{i}-x_{i}^{\prime }\right\|}^{2}$$

where $x_{i}$ and $x_{i}^{\prime }$ represent the actual and reconstructed genotype values for sample  i, and n is the total number of samples with i∈[1,n]. This effectively computes the reconstruction error per one-hot encoded element across all samples.

The encoded and divided genotype data was then used to train the deep autoencoder. The dataset was split into training (60%), testing (20%), and validation (20%) sets using the train_test_split function from scikit-learn [11]. To optimize the performance of the compression model, we utilized the KerasRegressor wrapper from Keras and applied scikit-learn’s RandomizedSearchCV to tune the hyperparameters of the autoencoder model. This approach ensured that the model’s architecture and parameters were fine-tuned for the best possible compression performance.

#### 2.1.3. Genomic Prediction

##### GBLUP

The genomic best linear unbiased prediction (GBLUP) model [2], which uses the genomic relationship matrix, was employed to estimate GEBVs for all genotyped individuals. The model was expressed as:

(6) y=Xb+Za+e

where y was the vector of observed phenotypic values, b represented the vector of fixed effects (including the overall mean), and X was the incidence matrix that links the fixed effects to the phenotypic values. The vector a contained the breeding values, which were assumed to follow a normal distribution $N(0,\;G\sigma_{a}^{2}$), where $\sigma_{a}^{2}$ was the additive genetic variance and G was the genomic relationship matrix. The matrix X connected the breeding values a to the phenotypic values y. The vector e represented random errors, assumed to follow a normal distribution $N(0,\;I\sigma_{e}^{2}$), where I was the identity matrix and $\sigma_{e}^{2}$ denotes the residual variance. For WGS data, G was constructed using all markers as follows [2]:

(7) $$G=\frac{WW^{T}}{\sum 2p_{j}q_{j}}$$

where $p_{j}$ was the alternative allele frequency at locus j, $q_{j}=(1-p_{j})$, W was the centered marker matrix where genotypes were subtracted 2$p_{j}$. For genotype data compressed using a deep autoencoder, the calculation of the G was expressed as:

(8) $$G=\frac{XX^{T}}{n}$$

where X was the standardized genotype matrix obtained after compression by the deep autoencoder, where each row represented an individual and each column represented a genetic marker. n was the number of markers (i.e., the number of columns in the genotype matrix).

##### Bayesian and Machine Learning Methods

The high dimensionality of WGS data, often comprising millions of markers, posed significant computational challenges for the direct application of Bayesian and machine learning methods. However, deep autoencoder compression reduced the genotype matrix to approximately 50 K markers, mitigating computational limitations while retaining essential genetic information. This dimensionality reduction enabled the efficient use of Bayesian and machine learning models with minimal loss of genetic variation, ultimately enhancing the scalability and accuracy of genomic predictions.

The Bayesian methods evaluated in this study included BayesA [1], BayesB (with π set to 0.99) [1], BayesCpi [12], and BayesLasso [13]. For these methods, MCMC were run for 18,000 cycles of Gibbs sampling, with the first 3000 cycles discarded as burn-in. The machine learning methods assessed are support vector regression (SVR) [14], random forest (RF) [15], kernel ridge regression (KRR) [16] and extreme gradient boosting (XGB) [17]. Hyperparameter optimization for machine learning models was performed through grid search, identifying the optimal parameter combinations. All hyperparameter tuning was conducted using fivefold cross-validation to ensure robust model evaluation. Bayesian methods were implemented using the BGLR R package (version 4.3.2) [18], while machine learning models were developed using the scikit-learn package for Python (version 3.8.3) [11].

### 2.2. Evaluation of the Performance of the DAGP Method

#### 2.2.1. Sturgeon Dataset

The Russian sturgeons used in this study were sourced from Hangzhou Qiandaohu Xunlong Sci-tech Co., Ltd. (Hangzhou, China) [19]. In 2012, six female and twenty-six male sturgeons were artificially inseminated, producing twenty-six full-sibling families. At eight years of age, the developmental status of fish roe was assessed, and fish with an average roe diameter greater than 2.8 mm were tagged and sampled for genomic analysis. These tagged fish were later processed for caviar production, and data on body weight, total caviar weight, and caviar color were recorded. Caviar color was subjectively scored on a scale from 1 to 4, based on color depth. A total of 673 fish with recorded phenotypes were selected for analysis. As all fish were reared under the same environmental conditions, the fixed effects in Equation (2) only included the overall mean. Genomic DNA was extracted, and WGS was performed using the BGI-T7 platform with 150 bp paired-end reads. Raw reads were filtered for quality, and sequencing data were aligned to the sterlet (*Acipenser ruthenus*) reference genome [20]. SNPs were called using GATK (version 3.5) [21]. Following SNP calling, missing genotypes were imputed using Beagle (version 5.1) [22]. Rigorous quality control was applied to exclude SNPs with a call rate below 90%, a minor allele frequency (MAF) under 0.05, and those with significant deviations from Hardy–Weinberg equilibrium (*p* < 10^−7^). The final dataset for analysis contained 10,409,793 SNPs. The sturgeon genotype data underwent eight levels of deep autoencoder compression (Net 1–Net 8), with dimensionality reduced from 10 million (Net 1) to 5000 markers (Net 8).

#### 2.2.2. Maize Dataset

This study included 350 elite maize inbred lines [23], consisting of both US and Chinese lines. These lines were selected based on published literature, pedigrees, hybrid registration information, and breeder communications. The lines were planted and phenotyped across four environments over two consecutive years: Langfang, Hebei (LF2016 and LF2017), Ledong, Hainan (HN2016), and Gongzhuling, Jilin (JL2017). All trials were conducted using a randomized complete block design, with one replicate in LF2016 and HN2016, and two replications in LF2017 and JL2017. For phenotyping, at least five plants per plot were measured for each line. The following traits were recorded: (1) DTA: Days to anthesis, (2) EP: Relative ear height, and (3) TBN: Tassel branch number. Least square means analysis was carried out for each trait, with genotype and environment were treated as fixed effects. The corrected phenotypes were then used as the phenotype values for genomic prediction. Genotypic data for the 350 inbred lines were obtained through DNA extraction and sequencing. Six inbred lines (Mo17, Zheng58, Chang7-2, 478, 5003, and 8112) were previously sequenced to over 20× coverage, and the remaining 344 lines were genotyped using Illumina X-ten sequencing (Illumina Inc., San Diego, CA, USA), generating approximately 69.3 billion 150 bp paired-end reads. After quality control, SNPs with a call rate below 90%, a MAF under 0.05, or significant deviations from Hardy–Weinberg equilibrium (*p* < 10^−7^) were excluded. As a result, a total of 2,663,873 SNPs were retained for genomic prediction analysis. The maize genotype data were subjected to seven levels of deep autoencoder compression (Net 1–Net 7), with dimensionality progressively reduced from 2 million (Net1) to 5000 markers (Net 7). Descriptive statistics for the analyzed traits in the sturgeon and maize datasets are shown in Table 1.

#### 2.2.3. Assessing Prediction Efficiency

To evaluate the predictive performance of genomic models, we employed a 20-repetition 5-fold cross-validation (CV) approach. Prediction accuracy was assessed by calculating Pearson’s correlation coefficient between the observed phenotypic values (y) and the GEBV for the validation set, as expressed by:

(9) $$r\left(y,GEBV\right)=\frac{Cov\left(y,GEBV\right)}{\sqrt{Var\left(y\right)*Var\left(GEBV\right)}}$$

where Cov(y,GEBV) is the covariance between y and GEBV, and $Var\left(y\right)$ and $Var\left(GEBV\right)$ are the variances of y and GEBV, respectively. The regression coefficient of y on GEBV was used to measure prediction bias, which was quantified as the absolute deviation of the regression coefficient from 1, it can be written as:

(10) $$Bias=\left|1-\frac{Cov\left(y,GEBV\right)}{Var\left(GEBV\right)}\right|$$

where $\left|\cdot \right|$ represents the absolute value. Additionally, model performance was compared using the mean squared error (Mse) and mean absolute error (Mae), calculated as:

(11) $$Mse=\frac{1}{n}\sum_{i=1}^{n}{\left(y_{i}-{GEBV}_{i}\right)}^{2}$$

(12) $$Mae=\frac{1}{n}\sum_{i=1}^{n}\left|y_{i}-{GEBV}_{i}\right|$$

where n is the number of individuals in the validation set, and $y_{i}$ and ${GEBV}_{i}$ represent the y and GEBV for individual i, respectively.

## 3. Results

### 3.1. Compression of Whole-Genome Sequencing Data

Table 2 summarizes the performance of deep autoencoder architectures in compressing WGS data from both the Sturgeon and Maize datasets. Each model was characterized by a specific network architecture (number of neurons per layer), batch size, number of epochs, number of split files, and dimension (marker density). Compression performance was evaluated in terms of mean squared error (MSE) and compression ratio (%). For sturgeon, higher marker densities were associated with lower compression ratios. Net 1 (312, 250, 200, 100 neurons) achieved a 3.94% compression ratio at a marker density of 10 million, maintaining an MSE of 0.027. Conversely, a more compact model (Net 8, 10, 8, 6, 5 neurons) achieved a significantly higher compression ratio (99.95%) at a lower marker density (5000), although with a slightly increased MSE of 0.113. A similar pattern was observed in Maize, where Net 1 (79, 63, 50, 20 neurons) compressed 2 million markers to 24.92% of the original size with an MSE of 0.036, while the smallest model (Net 7, 10, 8, 6, 5 neurons) achieved a 99.81% compression ratio at a marker density of 5000, with an MSE of 0.123. These results highlight the potential of deep autoencoders for efficient WGS data compression with minimal information loss.

### 3.2. Performance Evaluation of DAGP

**Figure 2:**
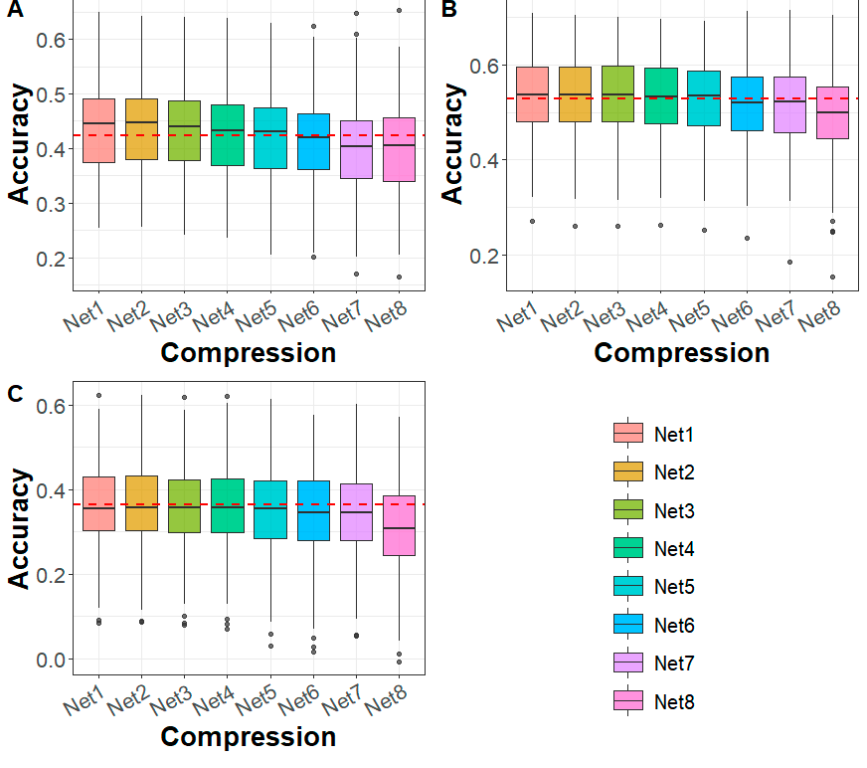
Genomic prediction accuracy for (**A**) CY, (**B**) CC, and (**C**) BW in sturgeon across different deep autoencoder compression scenarios from the first to the eighth compression (Net 1–Net 8, with marker densities ranging from 10,000,000 to 5000) using the GBLUP method. The red line represents the genomic prediction accuracy based on whole-genome sequencing data. CY: caviar yield, CC: caviar color, BW: body weight.

To assess the impact of deep autoencoder-based compression on genomic prediction, we conducted genomic prediction using compressed WGS data from the sturgeon and Maize datasets. In the sturgeon dataset, we evaluated genomic prediction accuracy for caviar yield, caviar color, and body weight traits after applying deep autoencoder-based compression (Figure 2). The results demonstrated a trend of flattening at first and then declining in genomic prediction accuracy from Net 1 (first compression) to Net 8 (eighth compression). At the fifth compression level (Net 5), with a marker density of 50 K and a compression ratio of 99.52%, the genomic prediction accuracy was consistent with that of the WGS markers. Remarkably, for compression levels Net 1 to Net 4, genomic prediction accuracy was higher than that of WGS markers, especially for traits like caviar yield, suggesting that moderate compression may even enhance the prediction of certain traits. However, for compression levels Net 6 to Net 8, the prediction accuracy fell below that of WGS markers. In terms of prediction bias, the results showed that in all cases, the prediction bias using compressed genomic data was lower than when using WGS (Figure S1). This indicates that compression helped reduce the bias in genomic prediction across all compression levels. Furthermore, the mean squared error (Mse) and mean absolute error (Mae) did not show significant differences between the compressed genomic data and those based on the WGS-based predictions (Figures S2 and S3).

**Figure 3:**
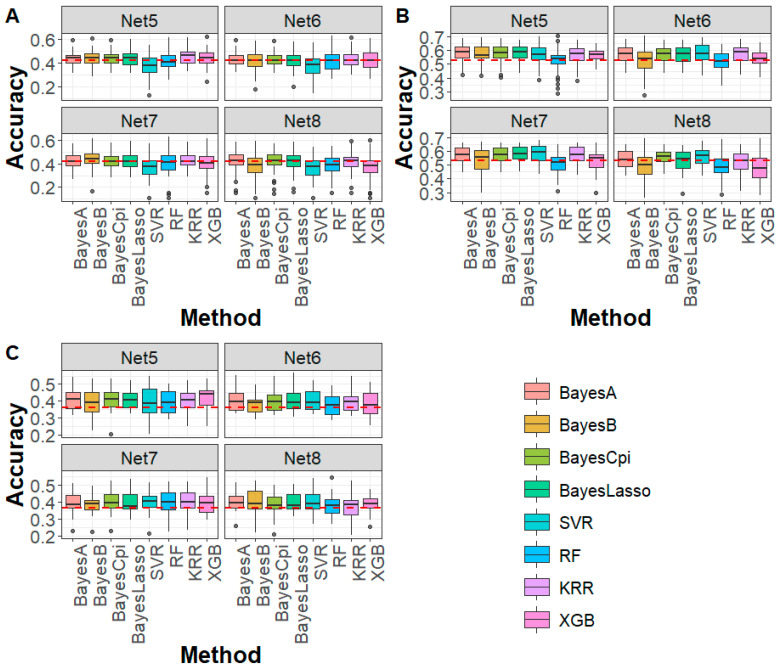
Genomic prediction accuracy for (**A**) CY, (**B**) CC, and (**C**) BW in sturgeon across different deep autoencoder compression scenarios from the fifth to the eighth compression (Net 5–Net 8, with marker densities ranging from 50,000 to 5000) using Bayesian and machine learning methods. The red line represents the genomic prediction accuracy based on the GBLUP method using whole-genome sequencing data. CY: caviar yield, CC: caviar color, BW: body weight.

As shown in Figure 3, for caviar yield, caviar color, and body weight traits in the sturgeon population, under Net 5 (marker density of 50 K, 99.52% compression ratio), all Bayesian methods outperformed the WGS-based GBLUP method. The average accuracy improvement was 1.42% for caviar yield, 4.82% for caviar color, and 4.57% for body weight. For machine learning methods, genomic prediction accuracy was generally higher than that of GBLUP, with an average improvement of 3.47%. However, for SVR and RF in caviar yield and RF in caviar color, the accuracy was slightly lower or comparable to that of GBLUP. Under Net 6 to Net 8 (marker density < 50 K), genomic prediction accuracy for most Bayesian and machine learning methods remained comparable or higher than the WGS-based GBLUP, except for caviar yield, where a slight decline in accuracy was observed. These findings indicate that despite some information loss, deep autoencoder-based compression effectively preserved key genetic signals, enabling robust genomic prediction. Notably, in most cases, the prediction bias of Bayesian and machine learning methods after deep autoencoder-based compression was comparable to that of WGS-based GBLUP (Figure S4). However, in specific cases, such as SVR, higher bias was observed in caviar yield. Furthermore, for both Mse and Mae, all Bayesian and machine learning methods consistently outperformed the WGS-based GBLUP (Figures S5 and S6), reinforcing the advantage of DAGP for genomic prediction using WGS data.

**Figure 4:**
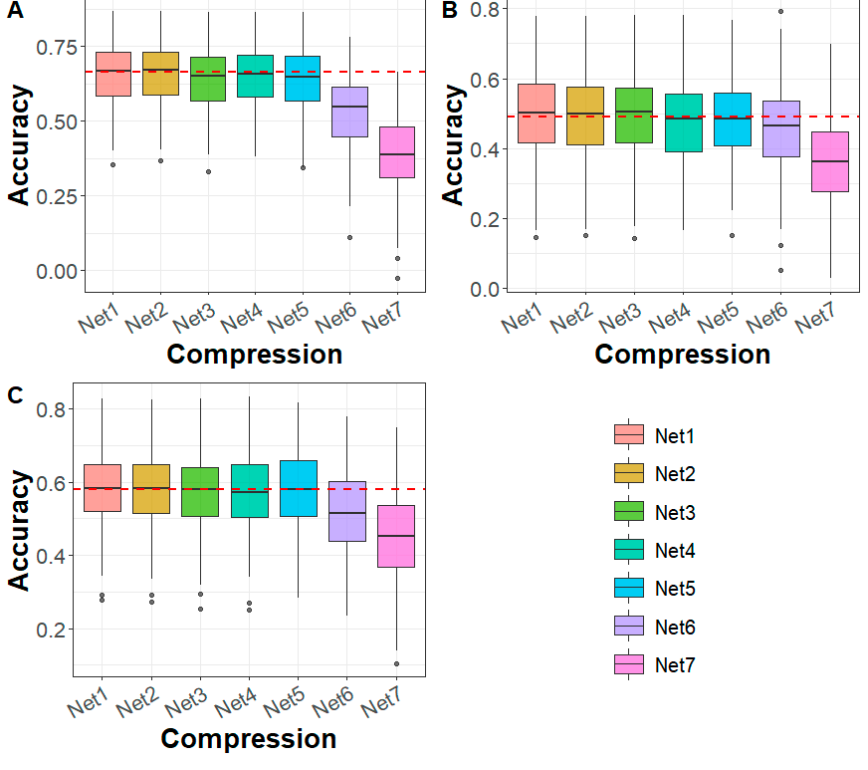
Genomic prediction accuracy for (**A**) DTA, (**B**) EP, and (**C**) TBN in maize across different deep autoencoder compression scenarios from the first to the seventh compression (Net 1 to Net 7, with marker densities ranging from 2,000,000 to 5000) using the GBLUP method. The red line represents the genomic prediction accuracy based on whole-genome sequencing data. DTA: days to anthesis, EP: the relative height of the ear, TBN: tassel branch number.

In the maize dataset, for DTA, EP, and TBN traits, the genomic prediction accuracy based on Net 1–Net 5 compression was comparable to that of WGS-based predictions (Figure 4). However, after Net 5 compression (Net 6–Net 7), prediction accuracy declined and consistently fell below that of the WGS-based predictions. Regarding prediction bias, for the DTA trait, the bias under Net 1 to Net 4 compression was higher than that based on WGS, while Net 5 to Net 7 showed similar or lower bias compared to WGS. For the EP and TBN traits, prediction bias was generally comparable to that of WGS-based predictions (Figure S8). In terms of Mse and Mae, the values remained consistent with WGS across all cases (Figures S9 and S10).

**Figure 5:**
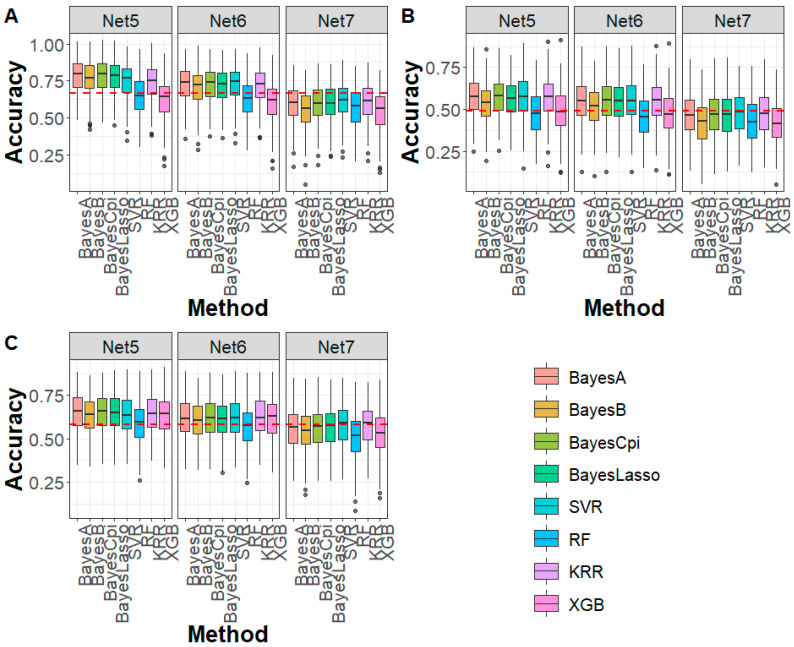
Genomic prediction accuracy for (**A**) DTA, (**B**) EP, and (**C**) TBN in maize across different deep autoencoder compression scenarios from the fifth to the seventh compression (Net 5 to Net 7, with marker densities ranging from 20,000 to 5000) using Bayesian and machine learning methods. The red line represents the genomic prediction accuracy based on the GBLUP method using whole-genome sequencing data. DTA: days to anthesis, EP: the relative height of the ear, TBN: tassel branch number.

For the DTA, EP, and TBN traits in the maize population (Figure 5), under Net 5 compression (marker density of 20 K, 99.25% compression ratio), all Bayesian methods exhibited higher prediction accuracy compared to the WGS-based GBLUP method, with average improvements of 11.40%, 7.37%, and 6.69% for DTA, EP, and TBN, respectively. For machine learning methods, SVR and KRR showed improvements of 7.83% and 7.98% in prediction accuracy for DTA and EP, while RF and XGB performed slightly worse than GBLUP. For TBN, all machine learning methods outperformed GBLUP, with an average increase of 4.49%. For Net 6 (marker density of 10 K, 99.62% compression ratio), the genomic prediction accuracy trend was similar to that of Net 5, with both Bayesian and machine learning methods generally outperforming the WGS-based GBLUP. However, for Net 7, prediction accuracy for DTA, EP, and TBN using both Bayesian and machine learning methods was lower than the WGS-based GBLUP method. Regarding prediction bias, except for the RF method, which exhibited higher prediction bias compared to the WGS-based GBLUP, Bayesian and machine learning methods showed similar prediction bias to GBLUP (Figure S11). In terms of Mse and Mae, in all cases, both Bayesian and machine learning methods produced lower values than GBLUP (Figures S12 and S13), further demonstrating the advantages of DAGP in genomic prediction.

## 4. Discussion

Genomic prediction often faces challenges due to the limited sample size relative to the large number of genetic variants, especially the millions of genetic markers generated by WGS data. Although Bayesian and machine learning methods have advantages in genomic prediction of complex traits by making more suitable assumptions or capturing nonlinear relationships in genetic markers, they remain impractical for genomic prediction with millions of genetic markers, especially when compared to BLUP-based methods. In this study, we introduce DAGP, an innovative genomic prediction model designed to overcome these challenges. The model incorporated a deep autoencoder compression process, which facilitated efficient storage and retrieval by performing one-hot encoding and data division on the genotypic data. Through this process, WGS data undergoes over 99% compression with minimal loss of genetic information, effectively solving the storage and subsequent computation issues caused by the large number of genetic markers. After compression, genomic prediction can be performed using Bayesian and machine learning methods, yielding higher prediction accuracy than the WGS-based GBLUP method. We systematically evaluated the advantages of DAGP using datasets from animal (sturgeon) and plant (maize) species.

In genomic prediction methods, Bayesian methods typically assume specific distributions for the variables, requiring the establishment of prior distributions and the calculation of posterior distributions using Bayesian theorem [1]. This process involves methods like MCMC, including Gibbs sampling or Metropolis-Hastings (MH) sampling, which often require tens of thousands of iterations to achieve convergence. Each iteration reassesses all marker effect values in a sequential, non-parallel process, leading to substantial computational demands. Given the millions of markers in WGS data, direct application of Bayesian methods remains impractical. Numerous studies have demonstrated that Bayesian models achieved prediction accuracy comparable to or higher than the GBLUP model at moderate SNP densities [24,25,26,27,28]. However, their application to WGS data has been limited due to computational time and storage constraints. Machine learning methods have emerged as powerful tools for improving the accuracy of genomic prediction for complex traits [3]. Unlike traditional statistical models, machine learning methods can capture nonlinear relationships and high-order interactions among genetic markers. They are especially beneficial for high-dimensional genomic data, as they do not rely on strong assumptions about genetic architecture [29]. However, machine learning methods rely heavily on hyperparameter tuning, which significantly impacts their performance [28,30]. Optimal hyperparameter selection requires extensive computational resources and cross-validation strategies, making the process time-consuming. This challenge is further exacerbated at the WGS level, where millions of markers must be processed simultaneously. The high-dimensionality of WGS data increases computational demands, necessitating dimensionality reduction techniques to enable efficient model training and prediction.

Deep learning technology has been increasingly applied to genomic data compression for high-dimensional analysis. Nazzicari and Biscarini [31] proposed a dimensionality reduction method using the kinship matrix to compactly represent SNP genotype data. They stacked multiple kinship matrices and processed them using a 2D convolutional neural network for genomic prediction. Similarly, Kick et al. [32] applied principal component analysis for dimensionality reduction, integrating the processed data into a multimodal deep learning model, achieving performance comparable to GBLUP. While these approaches effectively reduced dataset dimensionality, they may also eliminate fine-grained genetic information, leading to inconsistent prediction accuracy. To address this challenge, we developed DAGP, a genomic prediction model that employs deep autoencoder compression. This method significantly reduced the dimensionality of WGS data while preserving essential genetic information. In both sturgeon and maize datasets, the prediction accuracy of GBLUP using deep autoencoder-compressed data remained comparable to that of uncompressed WGS data until marker densities dropped to 50 K and 30 K, respectively (Figure 2 and Figure 4). To further assess the effectiveness of deep autoencoder compression, we calculated the correlation between the upper triangular elements of the genomic relationship matrix at different compression levels (Net 1–8) and those from WGS data. In both species, the correlation exceeded 0.96 (Figures S7 and S14), indicating that deep autoencoder compression retained nearly all genetic information while significantly reducing data complexity.

It is worth noting that, despite retaining most common genetic signals, some fine-scale information may be lost during compression. Rare variants with low minor allele frequency, structural variations, or complex haplotype patterns may not be fully captured in the compressed representation. Consequently, analyses that rely on these features could be affected, although the bulk of common variation and major genotype patterns remain well preserved. We also observed trait-specific differences in prediction accuracy following compression. Based on previous GWAS studies in sturgeon [33], body weight is influenced by several large-effect loci, making its genetic signal more robust to compression. In contrast, caviar yield is highly polygenic, controlled by numerous small-effect loci, which are more sensitive to any loss of fine-scale information during compression. These observations suggest that the maximum compression level without significant loss of prediction accuracy may depend on both the linkage disequilibrium (LD) structure of the population (i.e., the number of effectively independent SNPs) and the number of underlying causal variants. Populations or traits with fewer independent loci or stronger LD patterns can tolerate higher compression, whereas highly polygenic traits with many causal SNPs are more sensitive to loss of fine-scale information. These observations highlight how underlying genetic architecture can influence the effectiveness of dimensionality reduction, emphasizing the biological relevance of trait-specific considerations in genomic prediction. In addition, deep autoencoder models are sensitive to hyperparameter selection (e.g., network architecture, learning rate, batch size), and training on WGS data entails substantial computational costs. The generalizability of DAGP to species with markedly different genetic architectures remains to be further evaluated.

Previous studies using simpler approaches, such as linkage disequilibrium (LD)-pruned marker sets combined with GBLUP, demonstrated only modest improvements in prediction accuracy compared to full WGS datasets [34]. While LD pruning reduces redundancy and computational load, it may also remove informative markers, thereby limiting the ability to fully capture genetic variation. GWAS-based SNP selection can prioritize trait-associated markers and achieve moderate gains in prediction accuracy, but it still relies on pre-selected subsets and may miss nonlinear interactions among loci. In contrast, the deep autoencoder compression employed in DAGP preserves both linear and nonlinear genetic signals across all markers, enabling more accurate and robust genomic prediction. This highlights that DAGP not only achieves substantial dimensionality reduction but also preserves or enhances predictive performance relative to conventional marker pruning or GWAS-based strategies.

After deep autoencoder compression, WGS data can be effectively utilized for genomic prediction using Bayesian and machine learning methods. Our results showed that DAGP, which integrated Bayesian and machine learning methods, achieved higher genomic prediction accuracy than WGS-based GBLUP method in both the sturgeon and maize datasets (Figure 3 and Figure 5), demonstrating the advantages of DAGP. This is mainly because WGS data includes all genetic markers affecting traits, without relying on the LD between SNPs and QTLs, enabling the tracking of all QTLs influencing traits. Therefore, after deep autoencoder compression, the WGS data retains the genetic information of WGS, and Bayesian and machine learning methods are better at capturing the relationships between genetic markers and traits, leading to improved prediction accuracy compared to GBLUP. Currently, the deep autoencoder compression in the GADP method is not linked to phenotypes. While GADP can be applied to all traits, the data resulting from deep autoencoder compression still retains noise loci from the WGS data, which negatively impacts the accuracy of genomic predictions. For instance, in this study, the GBLUP accuracy based on Net 6-Net 7 was lower than that using uncompressed WGS data (Figure 2 and Figure 4). Future improvements could focus on selectively retaining rare or complex variants, integrating multi-omics data, prioritizing functionally relevant markers, and tailoring feature selection to specific traits, which may further enhance both accuracy and robustness of genomic prediction.

## 5. Conclusions

In conclusion, we introduced DAGP, a genomic prediction method that leverages deep autoencoder compression to efficiently handle large-scale whole-genome sequencing data. By reducing genotypic data size by over 99% while minimizing genetic information loss, DAGP addresses the storage and computational challenges associated with high-density genomic markers. Our results demonstrated that DAGP, integrating Bayesian and machine learning methods, outperformed WGS-based GBLUP, in terms of prediction accuracy, bias, mean squared error (Mse), and mean absolute error (Mae) in both sturgeon and maize datasets. Therefore, we recommend DAGP as an optimal tool for genomic prediction, particularly when handling large-scale genetic datasets, as it offers improved prediction performance and computational efficiency. In the future, DAGP is expected to significantly advance genomic prediction across various species.

## Figures and Tables

**Table 1:** Descriptive statistics for analyzed traits in sturgeon and maize datasets.

Datasets	Trait ^a^	Sample Size	Mean	Standard Deviation	Coefficient of Variation (%)
Sturgeon	CY	673	0.190	0.057	30.000
	CC	673	2.453	0.653	26.620
	BW	673	19.933	4.029	20.213
Maize	DTA	350	67.319	4.408	6.548
	EP	350	36.084	5.304	14.699
	TBN	350	10.207	4.441	43.511

^a^ CY: Caviar yield, CC: Caviar color, BW: Body weight, DTA: Days to anthesis, EP: the relative height of the ear, TBN: tassel branch number.

**Table 2:** Hyperparameters determined of deep autoencoder compression for sturgeon and maize datasets.

Datasets	Compression	Hyperparameters							
		Neurons	Batch Size	Epochs	Number of Split Files	Original Dimension (p)	Compressed Dimension (d)	MSE	Compression Ratio (%)
Sturgeon	Net 1	312, 250, 200, 100	52	200	100,000	10,409,793	10,000,000	0.027	3.94
	Net 2	100, 80, 64, 20	32	200	100,000	10,409,793	2,000,000	0.022	80.79
	Net 3	20, 16, 12, 5	32	200	100,000	10,409,793	500,000	0.110	95.20
	Net 4	50, 40, 30, 20	32	200	10,000	10,409,793	200,000	0.091	98.08
	Net 5	20, 16, 12, 5	32	200	10,000	10,409,793	50,000	0.124	99.52
	Net 6	50, 40, 30, 20	32	200	1000	10,409,793	20,000	0.098	99.81
	Net 7	20, 16, 12, 10	32	200	1000	10,409,793	10,000	0.105	99.90
	Net 8	10, 8, 6, 5	32	200	1000	10,409,793	5000	0.113	99.95
Maize	Net 1	79, 63, 50, 20	32	200	100,000	2,663,873	2,000,000	0.036	24.92
	Net 2	20, 16, 12, 5	32	200	100,000	2,663,873	500,000	0.060	81.23
	Net 3	50, 40, 30, 20	32	200	10,000	2,663,873	200,000	0.107	92.49
	Net 4	20, 16, 12, 5	32	200	10,000	2,663,873	50,000	0.130	98.12
	Net 5	50, 40, 30, 20	32	200	1000	2,663,873	20,000	0.129	99.25
	Net 6	20, 16, 12, 10	32	200	1000	2,663,873	10,000	0.124	99.62
	Net 7	10, 8, 6, 5	32	200	1000	2,663,873	5000	0.123	99.81

MSE: mean squared error, is used to evaluate the performance of a deep autoencoder compression model. Compression ratio: represents the percentage reduction in size after compression and is calculated by comparing the original dimension p and the compressed dimension d as follows: $Compression\;ratio\left(\%\right)=(1-\frac{d}{p})\times 100$. A higher value indicates greater compression efficiency.

## Data Availability

The genotype and phenotype data for maize are available at https://www.nature.com/articles/s41588-020-0616-3 (accessed on 10 February 2024). The genotype and phenotype data for sturgeon used in the current study are available from the corresponding authors upon reasonable request. The code for running the DAGP method along with an example dataset can be found at https://github.com/Songhailiang307/DAGP (accessed on 17 February 2025).

## Supplementary Materials

The following supporting information can be downloaded at: https://www.mdpi.com/article/10.3390/biology14111622/s1.
