# Supplementary material for: A Deep Autoencoder Compression-Based Genomic Prediction Method for Whole-Genome Sequencing Data
Source: Biology (Basel). 2025 Nov 19;14(11):1622. doi: 10.3390/biology14111622 (PMC12650564; doi:10.3390/biology14111622)
Supplement: Supplementary file 1 [file biology-14-01622-s001.zip › biology-3980910-supplementary.pdf]

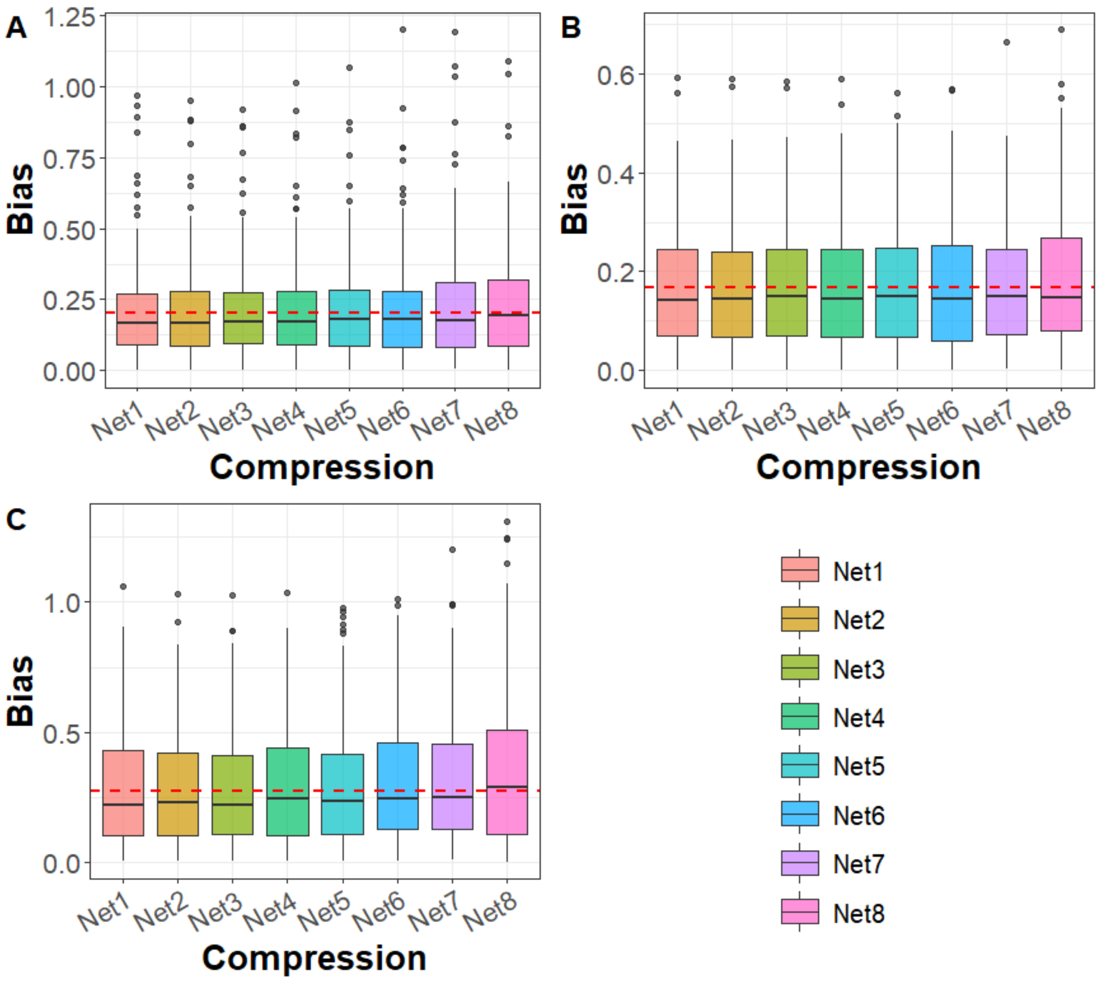

Figure S1 Bias of genomic prediction for (A) CY, (B) CC, and (C) BW in sturgeon across different deep autoencoder compression scenarios from the first to the eighth compression (Net1-Net8, with marker densities ranging from 10,000,000 to 5,000) using the GBLUP method. The red line represents the genomic prediction accuracy based on whole-genome sequencing data. CY: caviar yield, CC: caviar color, BW: body weight.

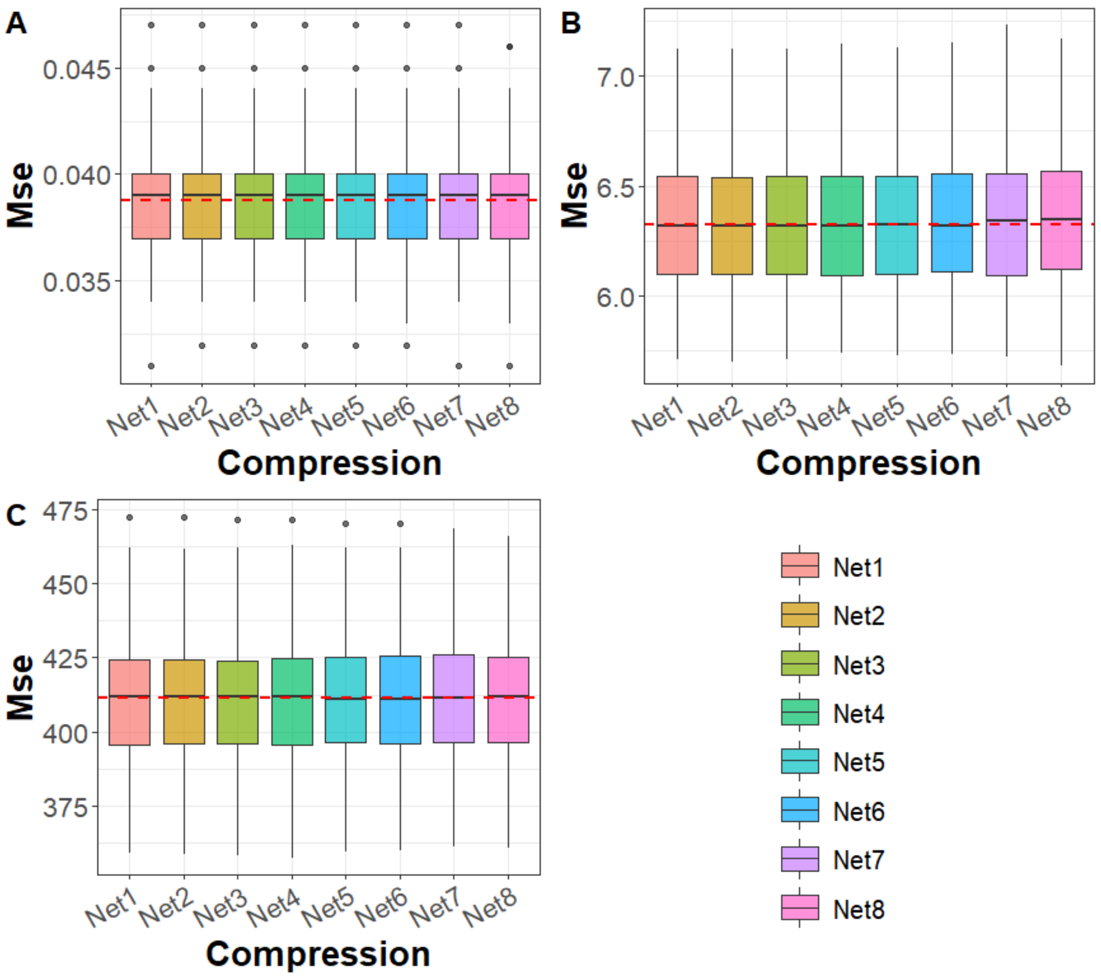

Figure S2 Mean squared error (Mse) of genomic prediction for (A) CY, (B) CC, and (C) BW in sturgeon across different deep autoencoder compression scenarios from the first to the eighth compression (Net1-Net8, with marker densities ranging from 10,000,000 to 5,000) using the GBLUP method. The red line represents the genomic prediction accuracy based on whole-genome sequencing data. CY: caviar yield, CC: caviar color, BW: body weight.

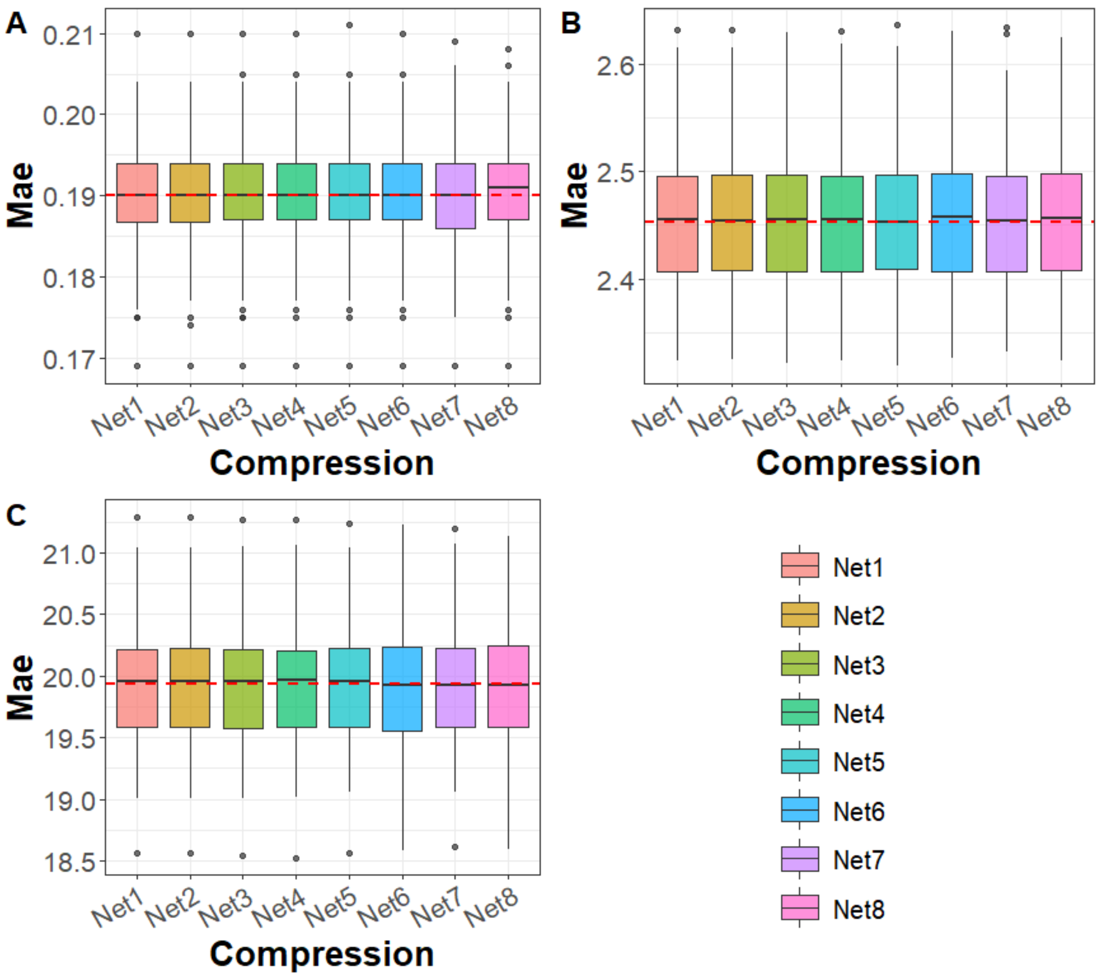

Figure S3 Mean absolute error (Mae) of genomic prediction for (A) CY, (B) CC, and (C) BW in sturgeon across different deep autoencoder compression scenarios from the first to the eighth compression (Net1-Net8, with marker densities ranging from 10,000,000 to 5,000) using the GBLUP method. The red line represents the genomic prediction accuracy based on whole-genome sequencing data. CY: caviar yield, CC: caviar color, BW: body weight.

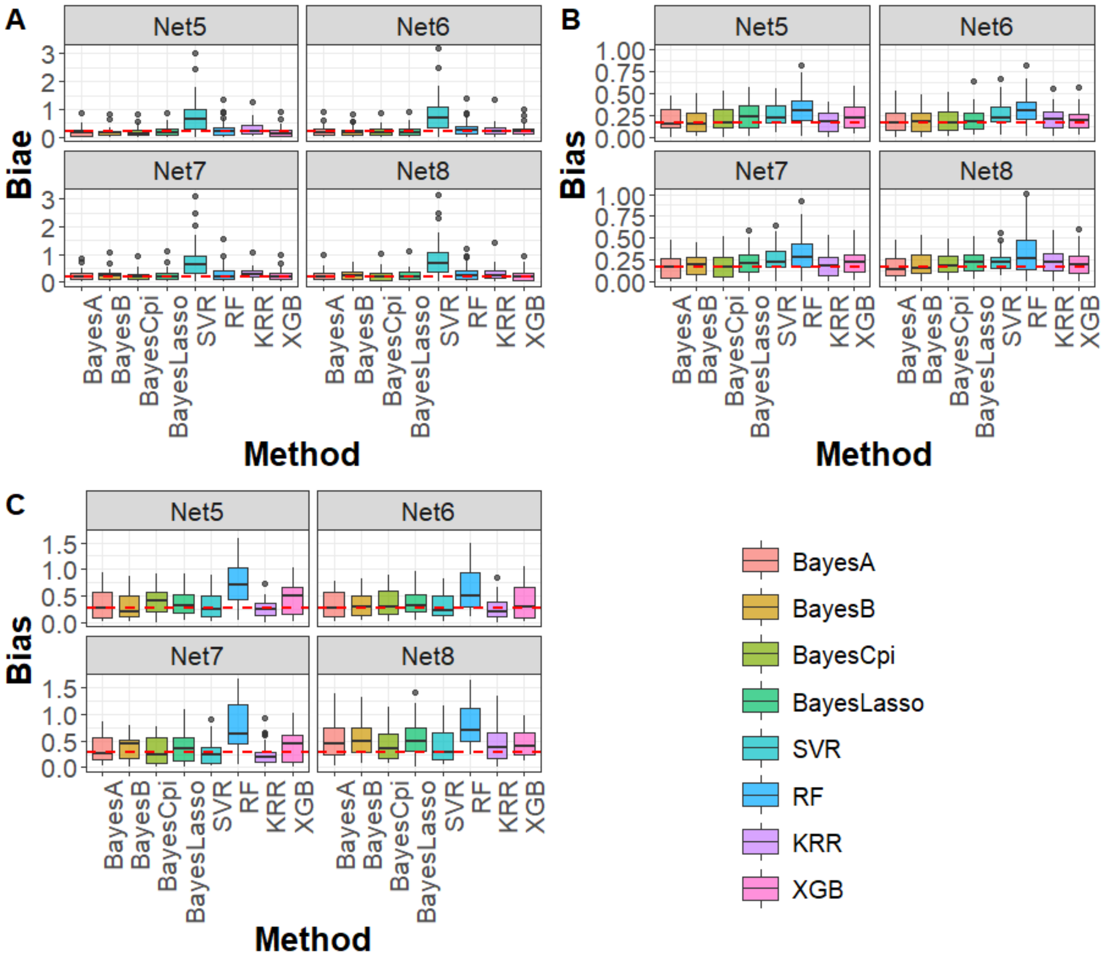

Figure S4 Bias of genomic prediction for (A) CY, (B) CC, and (C) BW in sturgeon across different deep autoencoder compression scenarios from the fifth to the eighth compression (Net5-Net8, with marker densities ranging from 50,000 to 5,000) using Bayesian and machine learning methods. The red line represents the genomic prediction accuracy based on the GBLUP method using whole-genome sequencing data. CY: caviar yield, CC: caviar color, BW: body weight.

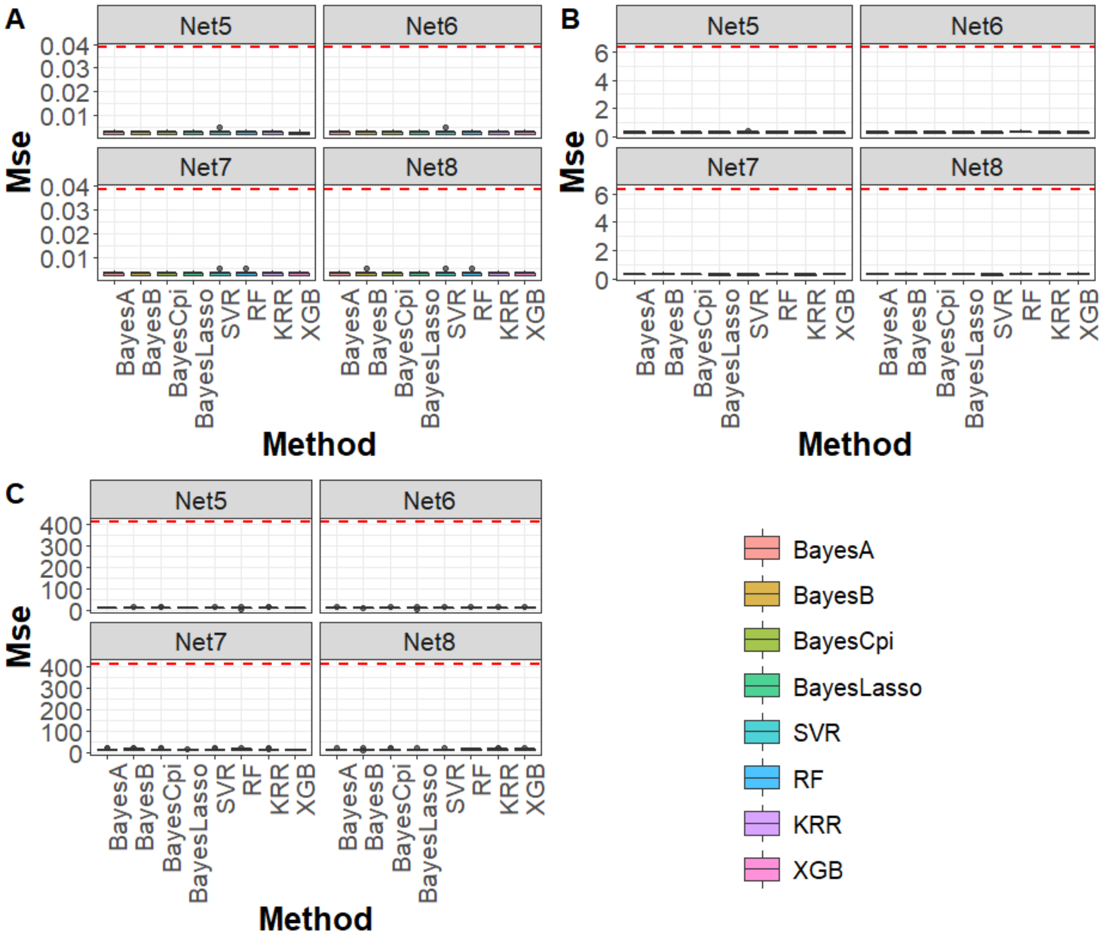

Figure S5 Mean squared error (Mse) of genomic prediction for (A) CY, (B) CC, and (C) BW in sturgeon across different deep autoencoder compression scenarios from the fifth to the eighth compression (Net5-Net8, with marker densities ranging from 50,000 to 5,000) using Bayesian and machine learning methods. The red line represents the genomic prediction accuracy based on the GBLUP method using whole-genome sequencing data. CY: caviar yield, CC: caviar color, BW: body weight.

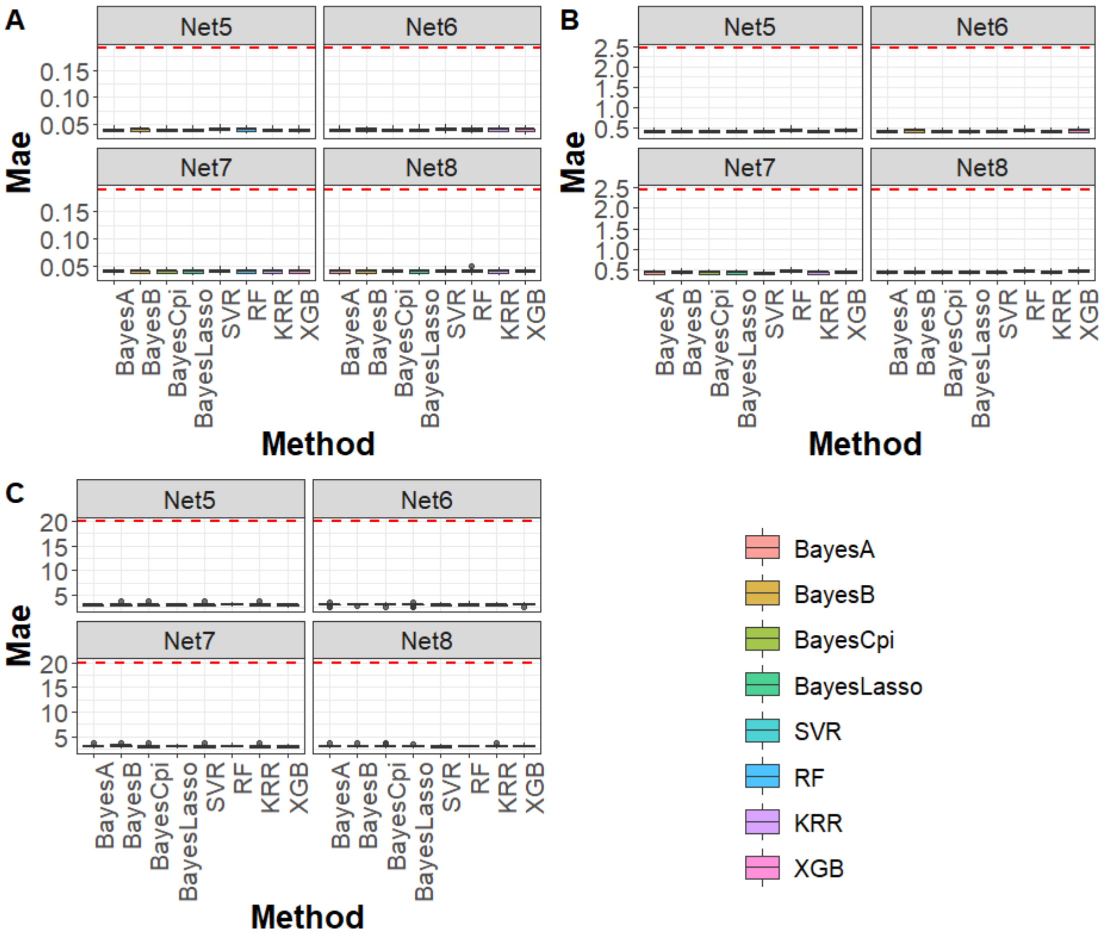

Figure S6 Mean absolute error (Mae) of genomic prediction for (A) CY, (B) CC, and (C) BW in sturgeon across different deep autoencoder compression scenarios from the fifth to the eighth compression (Net5-Net8, with marker densities ranging from 50,000 to 5,000) using Bayesian and machine learning methods. The red line represents the genomic prediction accuracy based on the GBLUP method using whole-genome sequencing data. CY: caviar yield, CC: caviar color, BW: body weight.

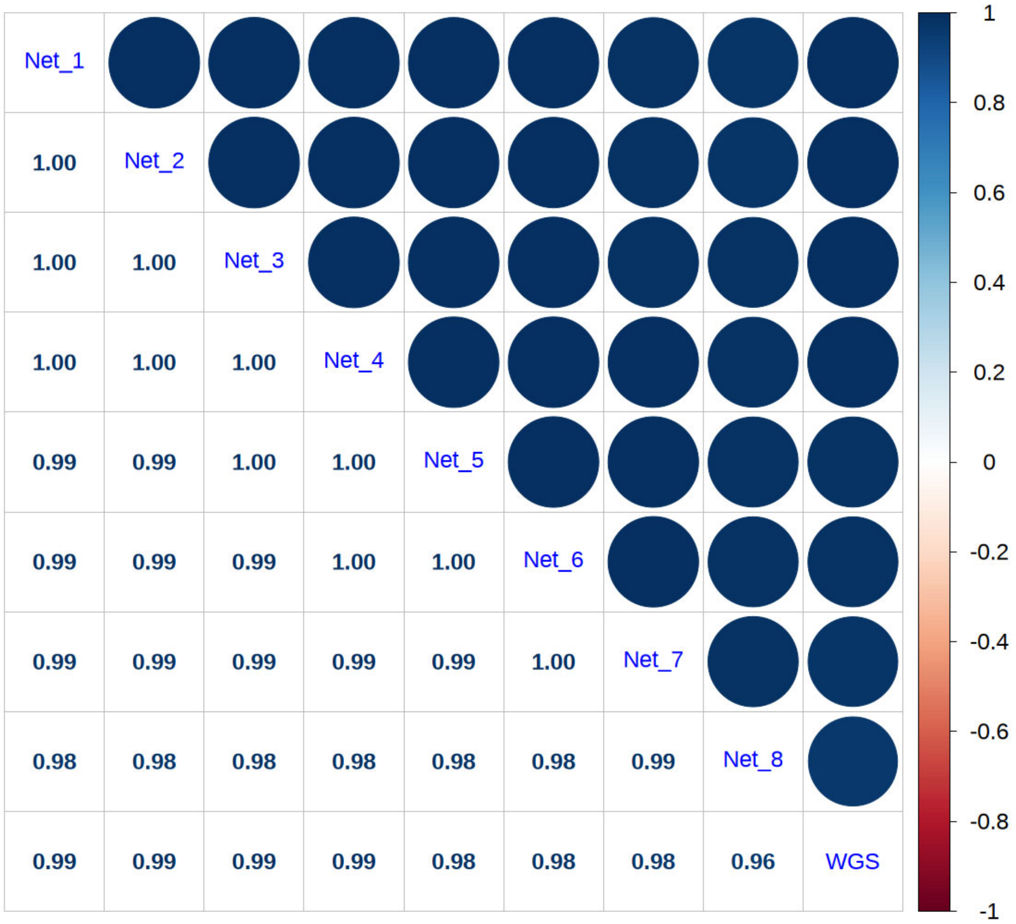

Figure S7 Correlation of the upper off-diagonal elements of different relationship matrices for sturgeon data under various compression levels (Net1–Net8) and whole-genome sequencing (WGS) data.

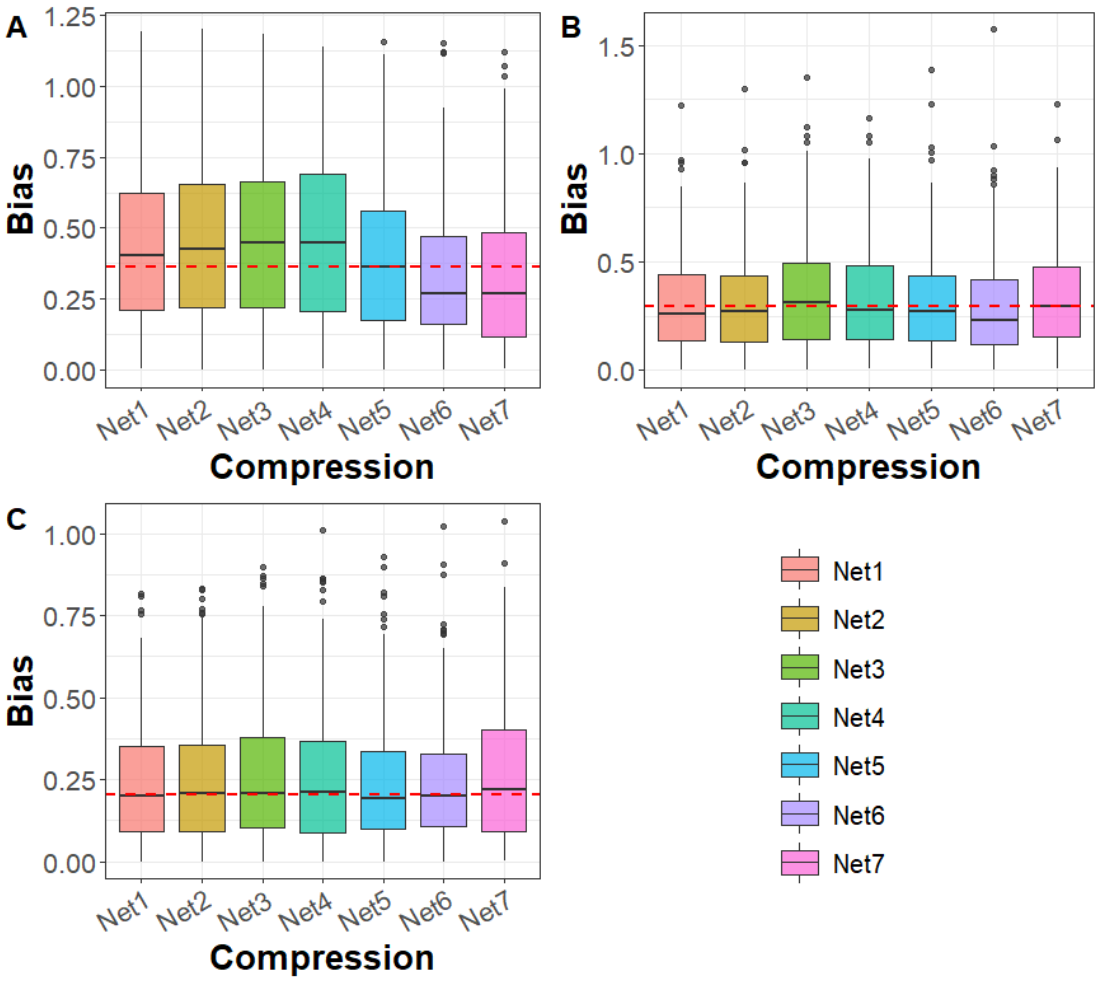

Figure S8 Bias of genomic prediction for (A) DTA, (B) EP, and (C) TBN in maize across different deep autoencoder compression scenarios from the first to the seventh compression (Net1 to Net7, with marker densities ranging from 2,000,000 to 5,000) using the GBLUP method. The red line represents the genomic prediction accuracy based on whole-genome sequencing data. DTA: days to anthesis, EP: the relative height of the ear, TBN: tassel branch number.

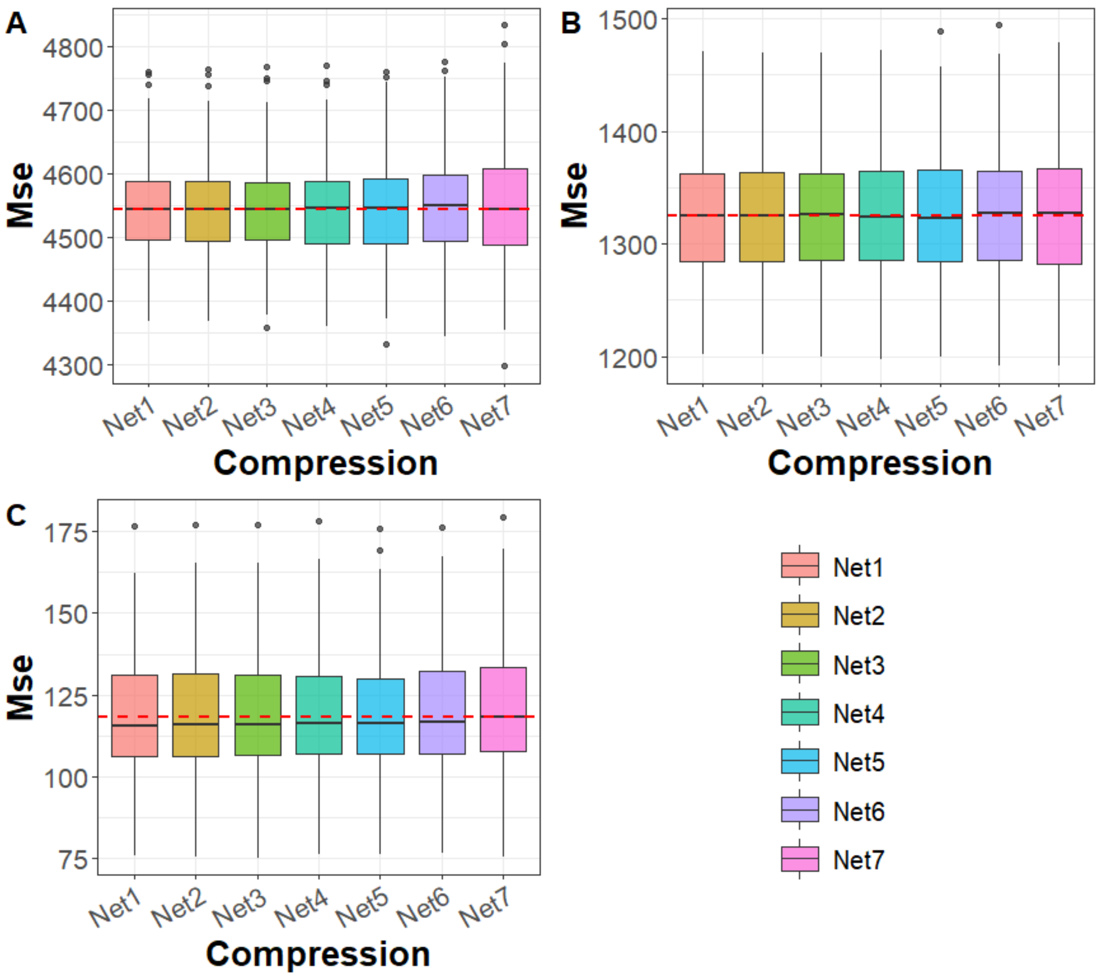

Figure S9 Mean squared error (Mse) of genomic prediction for (A) DTA, (B) EP, and (C) TBN in maize across different deep autoencoder compression scenarios from the first to the seventh compression (Net1 to Net7, with marker densities ranging from 2,000,000 to 5,000) using the GBLUP method. The red line represents the genomic prediction accuracy based on whole-genome sequencing data. DTA: days to anthesis, EP: the relative height of the ear, TBN: tassel branch number.

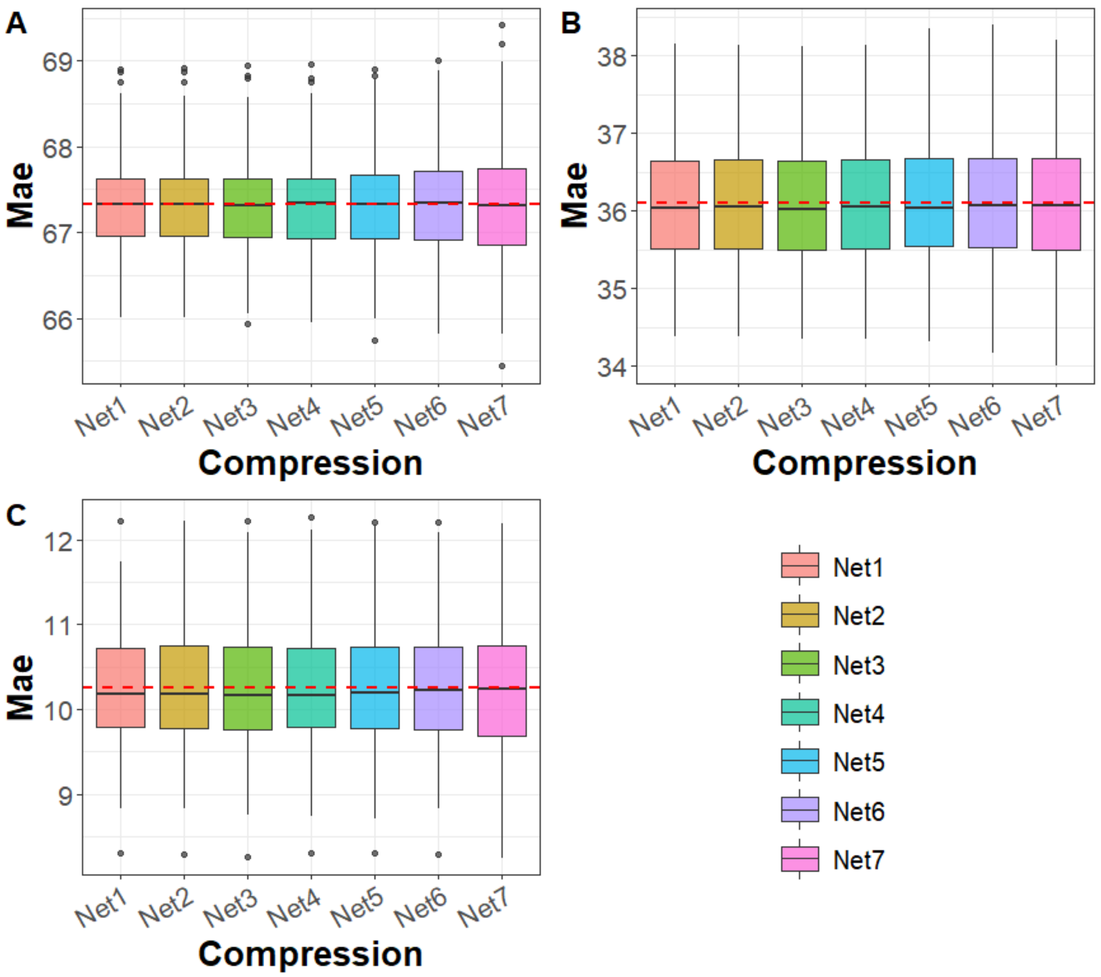

Figure S10 Mean absolute error (Mae) of genomic prediction for (A) DTA, (B) EP, and (C) TBN in maize across different deep autoencoder compression scenarios from the first to the seventh compression (Net1 to Net7, with marker densities ranging from 2,000,000 to 5,000) using the GBLUP method. The red line represents the genomic prediction accuracy based on whole-genome sequencing data. DTA: days to anthesis, EP: the relative height of the ear, TBN: tassel branch number.

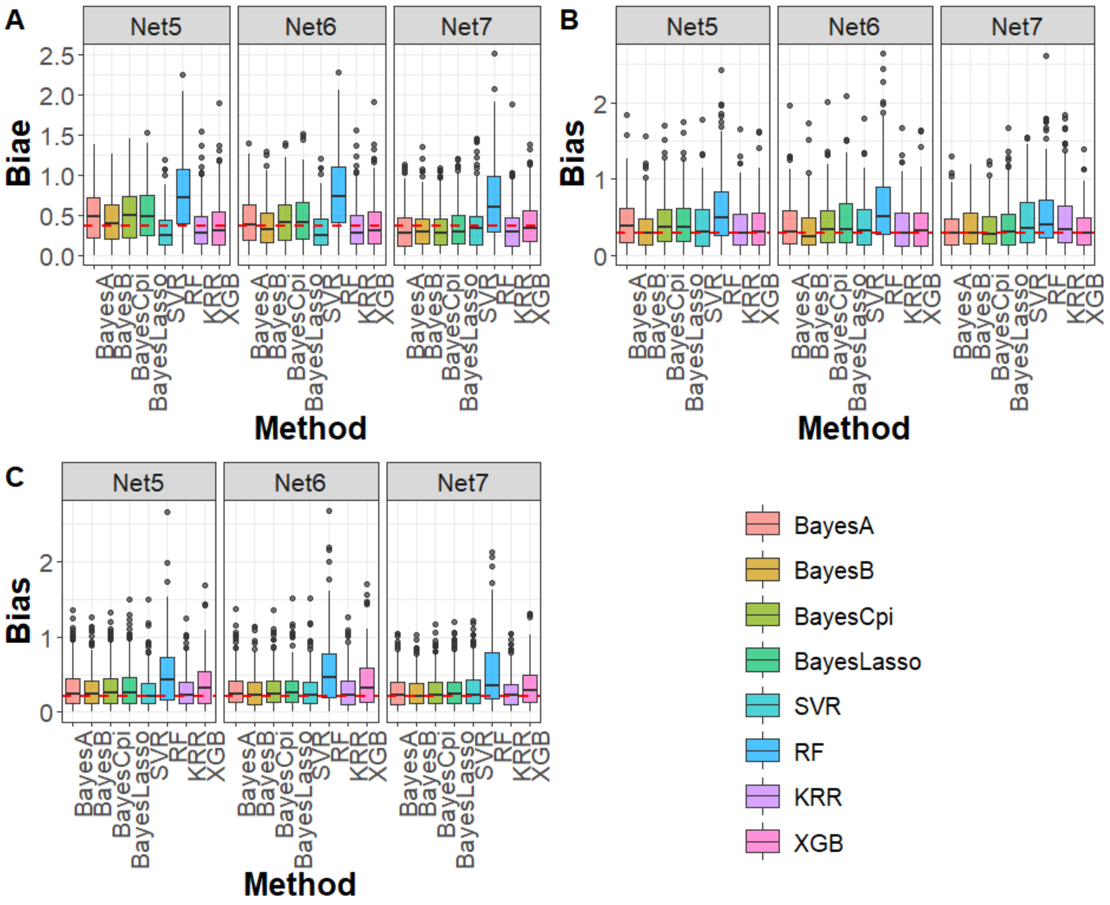

Figure S11 Bias of genomic prediction for (A) DTA, (B) EP, and (C) TBN in maize across different deep autoencoder compression scenarios from the fifth to the seventh compression (Net5 to Net7, with marker densities ranging from 20,000 to 5,000) using Bayesian and machine learning methods. The red line represents the genomic prediction accuracy based on the GBLUP method using whole-genome sequencing data. DTA: days to anthesis, EP: the relative height of the ear, TBN: tassel branch number.

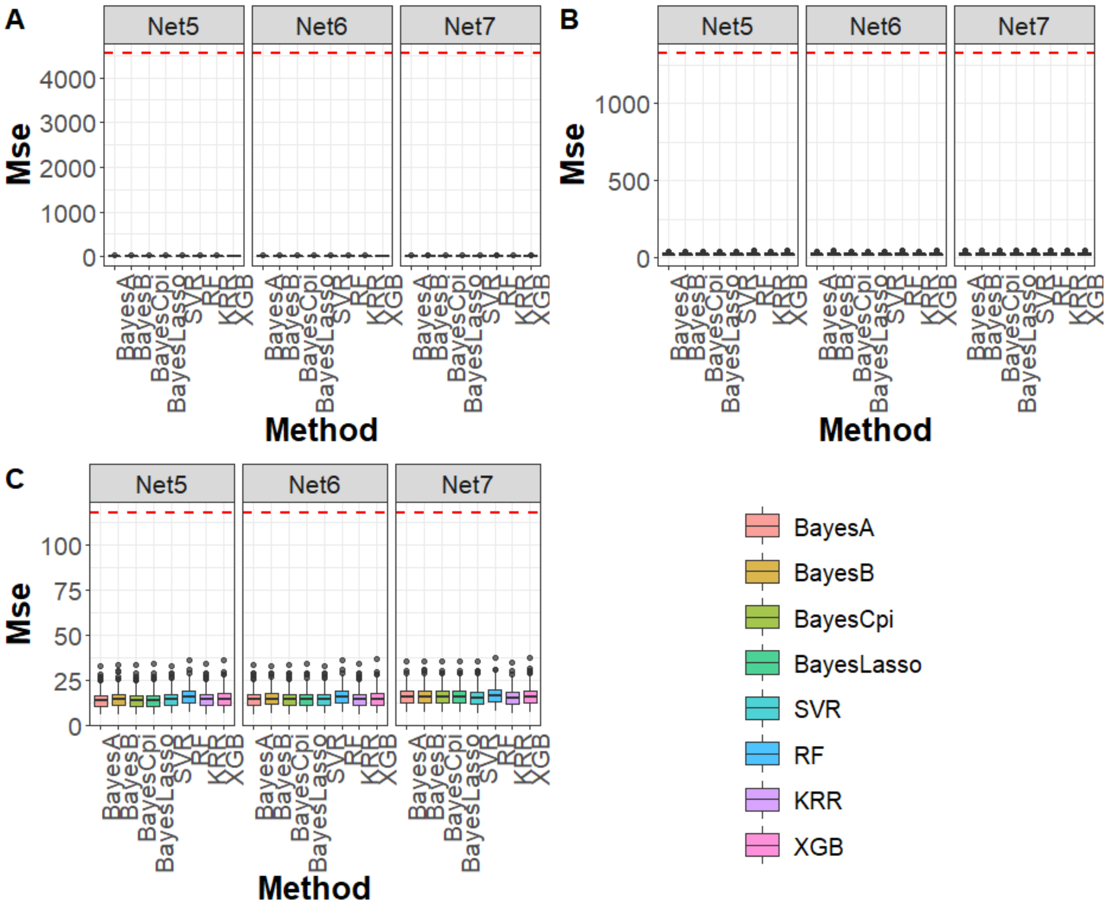

Figure S12 Mean squared error (Mse) of genomic prediction for (A) DTA, (B) EP, and (C) TBN in maize across different deep autoencoder compression scenarios from the fifth to the seventh compression (Net5 to Net7, with marker densities ranging from 20,000 to 5,000) using Bayesian and machine learning methods. The red line represents the genomic prediction accuracy based on the GBLUP method using whole-genome sequencing data. DTA: days to anthesis, EP: the relative height of the ear, TBN: tassel branch number.

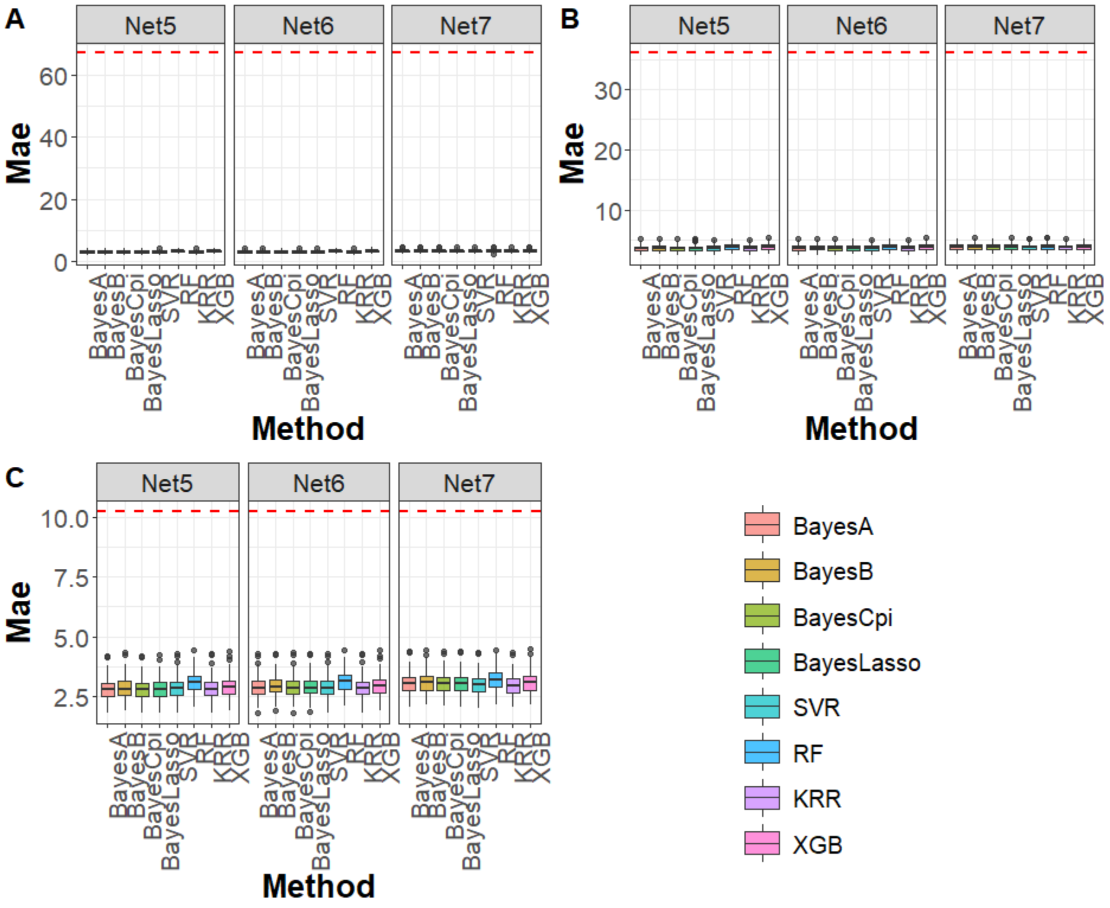

Figure S13 Mean absolute error (Mae) of genomic prediction for (A) DTA, (B) EP, and (C) TBN in maize across different deep autoencoder compression scenarios from the fifth to the seventh compression (Net5 to Net7, with marker densities ranging from 20,000 to 5,000) using Bayesian and machine learning methods. The red line represents the genomic prediction accuracy based on the GBLUP method using whole-genome sequencing data. DTA: days to anthesis, EP: the relative height of the ear, TBN: tassel branch number.

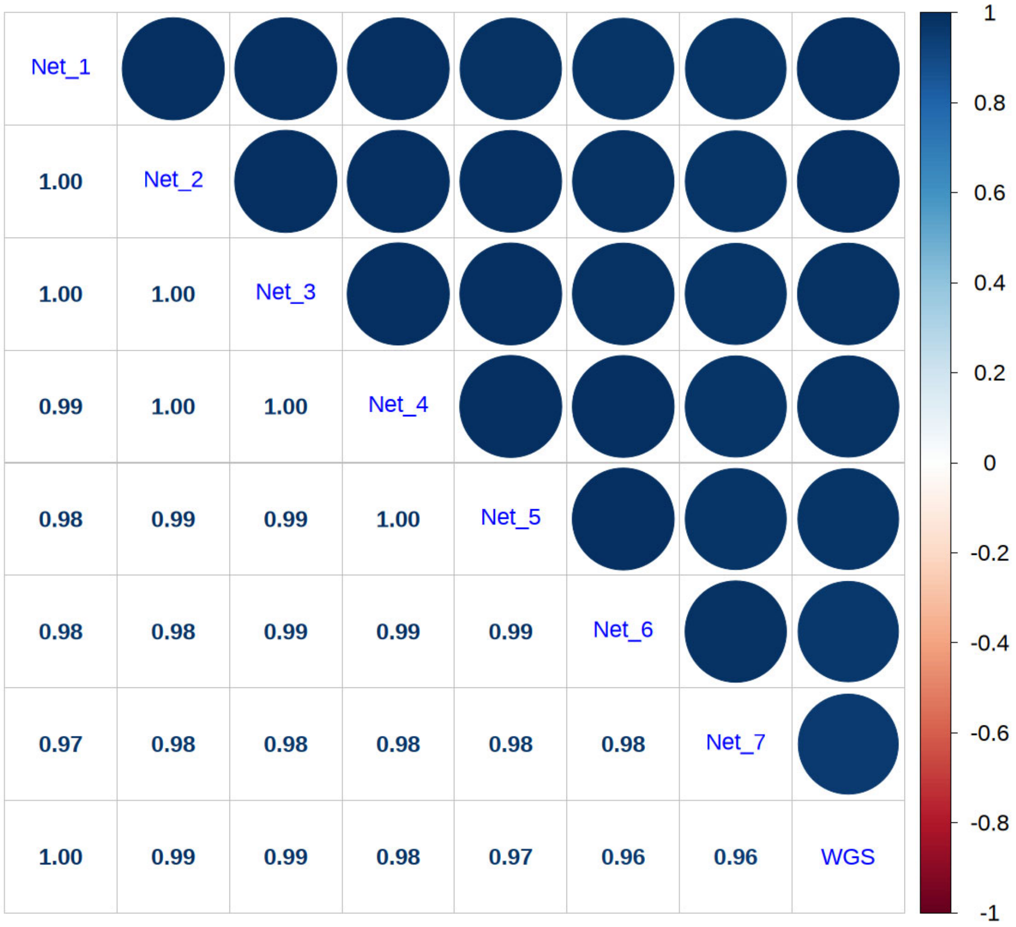

Figure S14 Correlation of the upper off-diagonal elements of different relationship matrices for maize data under various compression levels (Net1–Net7) and whole-genome sequencing (WGS) data.
